# Management of the paralyzed face using temporalis tendon transfer via intraoral and transcutaneous approach: Temporalis tendon transfer

**DOI:** 10.1186/s40902-018-0160-6

**Published:** 2018-09-05

**Authors:** Ji Yun Choi, Hyo Joon Kim, Seong Yong Moon

**Affiliations:** 1 Department of Otorhinolaryngology-Head and Neck Surgery, School of Medicine, Chosun University, 365, Pilmun-daero, Dong-gu, Gwangju, 501-759 South Korea; 2 Department of Oral and Maxillofacial Surgery, Chosun University Dental Hospital, 309, Pilmun-daero, Dong-gu, Gwangju, 501-759 South Korea; 3 Department of Oral and Maxillofacial Surgery, School of Dentistry, Chosun University, 309, Pilmun-daero, Dong-gu, Gwangju, 501-759 South Korea

**Keywords:** Facial nerve paralysis, Temporalis tendon transfer, Facial reanimation

## Abstract

Temporalis tendon transfer is a technique for dynamic facial reanimation. Since its inception, nearly 80 years ago, it has undergone a wealth of innovation to produce the modern operation. Temporalis tendon transfer is a relatively minimally invasive technique for the dynamic reanimation of the paralyzed face. This technique can produce significant and appropriate movement of the lateral oral commissure, more closely mimicking the normal side. The aim of this article is to review the technique of temporalis tendon transfer involving transferring of the coronoid process of the mandible with the insertion of the temporalis tendon via intra-oral and transcutaneous approach.

## Introduction

Long-standing facial paralysis may use reconstructive technique with neural reconstruction, muscle transposition, or transplantation [1]. Temporalis muscle transfer is one of the popular methods for reanimation for facial paralysis patient [2]. Minimally invasive temporalis tendon transfer technique is performed in patients with old facial nerve paralysis, which is mostly impossible to regenerate [3]. The success of treatment depends entirely on the function of the temporal muscle [4]. Measurement of myofunction can be easily identified by stimulating the bulging portion of the muscle when the muscle contracts. The purpose of the treatment of facial paralysis is to improve the symmetry of the face [5]. Therefore, it is a successful operation when the dynamic movement of the affected side is maximally symmetrical with the normal side. In this study, we evaluate the surgical technique of the temporalis tendon transfer via intraoral and transcutaneous approach and review the previous articles related to temporalis tendon transfer.

## Review

### Pertinent anatomy (Fig. 1)

The temporalis muscle is a fan-shaped muscle that is inserted into the coronoid process of the mandible from temporal fossa. The tendon starts from the upper part of the muscle in the form of a broad, thin layer, gradually becoming thicker and warming the coronoid process. The muscle has a sliding surface between the bone and muscle and passed through the inside arch of zygomatic bone. The temporal muscle tendon extends along the anterior aspect of the ramus of mandible, surrounding the medial and anterior surfaces of the coronoid process. A part of the temporal muscle tendon is found on the outer surface of the coronoid process. Most tendons are found on the inner surface of the coronoid process and extend downward toward the buccinator line. The coronoid process is directly accessible through the buccal space. This approach requires an understanding of the anatomy of the buccal space. The buccal space is laterally bounded by the superficial muscular aponeurotic system and facial muscles, medially bounded by buccinator muscle and buccal mucosa, and posteriorly bounded by the mandible and masseter muscles. The buccal space is mainly filled by buccal fat pads. Most facial nerves except the branch for buccinator muscle lie outside the buccal fat pads. Separating ligaments leading to the buccinator and temporalis muscle, delamination of the deep part of the buccal fat may expose the coronoid process.

**Fig. 1:**
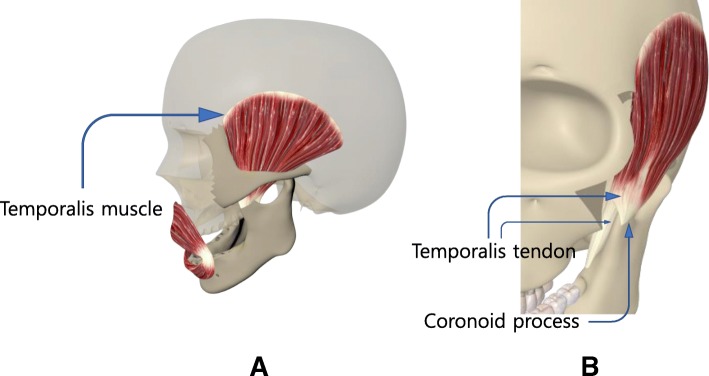
**a** Temporalis muscle and temporalis tendon, **b** Temporalis tendon and coronoid process

### Surgical technique

**Fig. 2:**
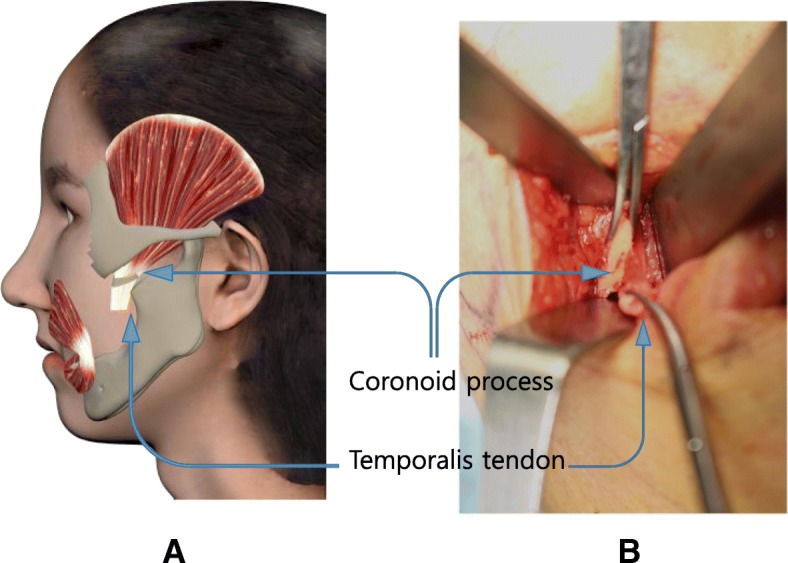
Separation of temporalis tendon from mandible ramus (**a** 3D simulation feature; **b** intraoperative view, separated temporalis tendon, and coronoid process)

Anterior ramus of the mandible and mandibular notch was exposed through the intra-oral vestibular incision. The coronoid process of the mandible is peeled and exposed, while taking care not to damage the medial temporalis tendon, and then, coronoid process was cut with electric saw obliquely. Temporalis tendon was separated from the medial side of the mandibular ramus. Skin incision was made along the nasolabial fold, and then, tunnels were formed from the skin incision site to the anterior ramus of the mandible by blunt dissection through the buccal space. (Fig. 2).

**Fig. 3:**
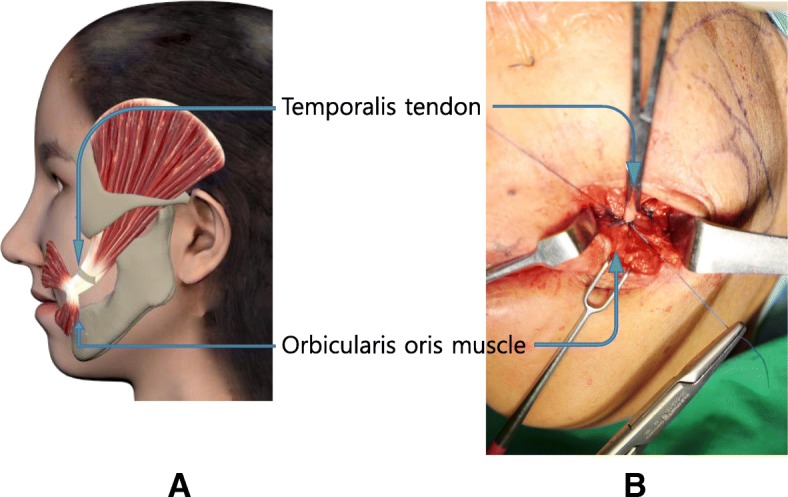
Fixation of temporalis tendon and coronoid process to orbicularis oris muscles. (**a** 3D simulation feature, **b** suture to the orbicularis oris muscle) (**a** transfer temporalis tendon with tip of coronoid process, **b** suture to the orbicularis oris muscle)

**Fig. 4:**
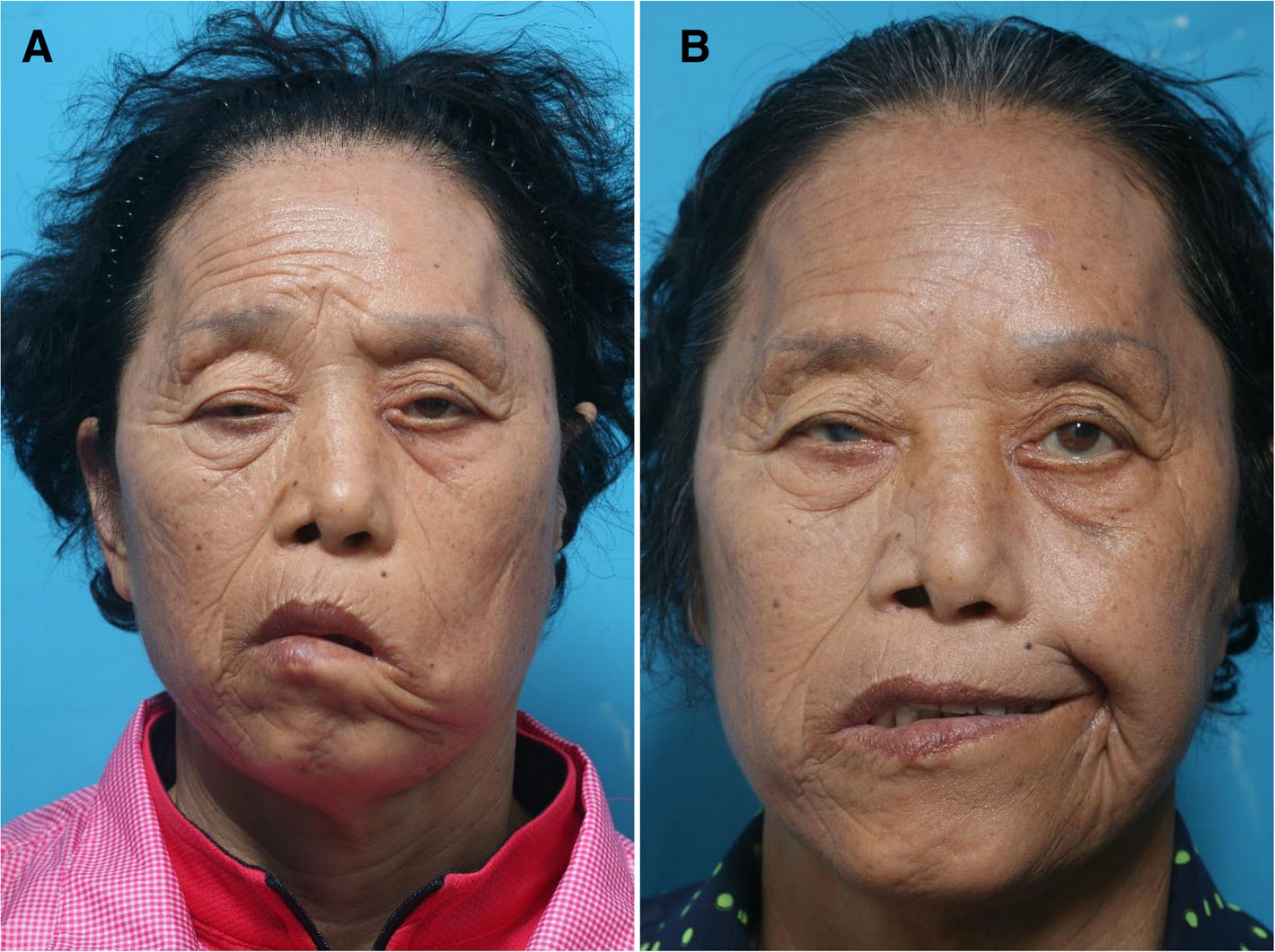
Clinical photos (pre-operation **a**, 6 months after operation **b**)

Separated coronoid process and temporalis tendon were moved to the lateral oral commissure through the buccal space, and fixed sutures were performed using 3–0 nylon at 3 points to the orbicularis oris muscles near the modiolus (Fig. 3). The skin and intraoral incision sites were sutured. During the 6 months after operation, she showed favorable results without any complications (Fig. 4).

In 1934, Gillies reported the temporalis muscle rotated downward at the zygoma to reach the lip subcutaneously to induce rehabilitation of the facial paralysis [6]. However, this resulted in significant muscle bulging over the zygoma and a residual hollow in the temporal fossa. McLaughlin first described the transoral technique for transferring the coronoid process with the attached temporalis tendon to the corner of the mouth [7]. This procedure avoids the fullness over the zygomatic arch area and the temporal donor site depression that is produced by the turned-down temporalis muscle flap. Breidahl published a method to improve the technique published by Champion [8, 9]. This technique was cutting the part of the zygomatic arch and exposing the temporalis tendon and then fully releasing it to the deep layer in an anteroposterior direction [8, 9]. In addition, when the length of the temporal muscle was insufficient, it was extended to include fascia lata [8, 9]. In 1997, Labbe reported a new myoplasty technique which separates the temporalis muscle from the temporal fossa and allows lengthening by redistribution of muscular fibers to the detriment of the posterior third. This allows the transfer of the coronoid tendinous insertions onto the lips [10]. This technique preserves the two neurovascular supplies and does not distort the cheek [11]. Contreras-Garcia and Braga-Silva introduced a trans-temporal approach using an endoscope [12], and Croxson described a modified approach to the temporalis tendon through a nasolabial incision [13]. Table 1 summarized the surgical techniques of previously reported temporalis tendon transfer.

**Table 1:** Surgical techniques of previously reported temporalis tendon transfer

Author	Number of patients	Incision site	Average follow-up	Complications	Remarks
Boahene et al. [3]	17	Melolabial crease	3 weeks	None	Single small incision and minimal dissection Using fascia lata graft if required In patients without a melolabial crease, a transoral incision or approach is chosen.
Breidahl et al. [8]	7	Temporal, nasolabial groove	3 months	Thigh hematoma Skin tethering	Using facia lata graft Avoid coronoidectomy
Labbe et al. [11]	10	Temporal, nasolabial groove	48 months	None	Temporalis muscle lengthening Coronoidectomy Zygomatic arch can be sectioned
Contreras-García et al. [12]	2	Oblique temporal, paramedian frontal, Intraoral	3 weeks	None	Based on Labbe’s technique Using endoscopic approach
Erni et al. [19]	10	Temporal, nasolabial groove, intraoral	80 months	Unknown	Using technique of McLoughlin et al. [7] Intraoral coronoidectomy
Balaji et al. [20]	5	Temporal, nasolabial groove, intraoral	60 months	None	Using facia lata graft Intraoral coronoidectomy
Terzis et al. [21]	35	Temporal, intraoral	38 months	Alopecia hollowing of the infratemporal fossa	Mini-temporalis transfer with free muscle transfer (cross-facial nerve graft and free muscle graft)
Har-Shai et al. [22]	15	Temporal, nasolabial groove	3 months	Unknown	Using Labbe’s technique [11] Using intraoperative electrical stimulation for accurate positioning
Griffin et al. [23]	7	Melolabial crease, mental crease	Unknown	Wound infection on graft area	Using facia lata graft Connect to upper lip and lower lip
Petersson et al. [24]	3	Intraoral, small external skin incision	13 months	None	Using facia lata graft

The location of temporalis tendon is determined by the preoperative smile pattern. The tendons are unfolded to make the width, and the suture is performed. To ensure proper tension, muscle stimulators are used to stimulate the temporal muscles to determine the degree of tension of the ligaments. Once the ideal tension is identified, the suture is performed to the insertion point of the orbicularis oris muscles and the zygomaticus muscles. If excessive tension is applied to reach the angle of the mouth, muscle sagging will occur. In this case, ligament extension transplantation should be considered.

Since temporalis tendon grafting can pull up oral commissure only in one direction, it is necessary to reproduce the normal side smile patterns similarly without showing the upper and lower lip showing teeth. Oral commissure raised by the zygomaticus muscle can be a goal for facial training [14]. This exercise takes the form of “forced laughter” by connecting simple smile patterns on the normal side to the smile caused by temporal muscle contraction on the affected side, so that learning the smile of the temporalis muscle is required after operation [15, 16].

In some patients, the lips have to be pulled to the opposite side for compensatory contractions. In this case, often the fascia lata is fixed to the opposite lip through the center of the lips. This method passes the fascial lata extension connected to the temporalis tendon through the tunnel of the upper and lower lips. Fix the fascia lata extension to the center of the upper and lower lips and pull the philtrum and lower lips to the center.

Temporal muscle transfer is an effective and reliable method for revitalizing a dynamic face. Approach to the coronoid process through the oral or buccal space allows direct access to the temporalis tendon with minimal incision. It is also possible to preserve the sliding surface between arch of zygomatic bone and temporalis tendon. The temporalis tendon is removed from the inside of the ramus of mandible until it reaches the buccinator line. Osteotomy of coronoid process is performed by oblique projection to allow most of the ligaments to attach. In most patients, a length sufficient to reach the angle of mouth can be obtained [17]. When the anchor point moves away from oral commissure, favorable results could not be obtained. The temporalis tendon and the orbicularis oris muscle should be fixed in close proximity to oral commissure.

There are some drawbacks of the standard surgical technique, however. By removing the muscle from its fan-shaped origin on the squamous portion of the temporal bone, an unsightly defect is created in the temporal region. In addition, if the muscle is reflected in the typical manner over the arch of the zygoma, a soft tissue protrusion overlying the zygomatic arch is produced. Doubling the muscle on itself over the zygomatic arch alters the dynamics of contraction, which may result in ischemia and likely decreases the contractile forces that can be obtained. However, this procedure is relatively easy to perform. It provides symmetrical oral commissure elevation. The vector of pull is essentially ideal. There is no soft tissue depression produced in the temple and no tissue protrusion overlying the zygomatic arch. The advantages of this technique are the lack of facial scarring, minimal postoperative discomfort, and rapid healing of the buccal mucosa. In contrast to the external approach, facial soft tissue swelling and bruising is minimal. The technique is associated with quick postoperative recovery and minimal morbidity, without a scar on the face. Both esthetic and functional improvements can be achieved with this technique. Orthodromic temporalis tendon transfer is indicated in cases of long-standing facial paralysis and in cases of subacute facial paralysis in patients who desire a single-stage procedure with nearly immediate dynamic function. It is possible only in patients with intact trigeminal motor function. Patients selected for the orthodromic temporalis insertion transfer procedure were critically analyzed for their smile pattern [18]. The reinsertion site of the temporalis tendon was selected based on the pattern of dominant musculature in the patient’s smile (more horizontal zygomaticus major vs more vertical levator labii superioris alaeque nasi). Comprehensive patient-directed physical therapy is then initiated. The goal of the preoperative physical therapy is for the patient to better understand how to consciously coordinate individual muscle contraction to produce facial expression, particularly the smile. At this time, patients are educated about their smile pattern (“canine” vs “Mona Lisa”) and about how they can modify this to adapt to the type of smile expected after surgery.

## Conclusions

The temporalis tendon transfer through the intraoral approach is a suitable procedure for dynamic treatment of minimally invasive facial paralysis in treating long-term facial paralysis patients. This procedure can keep the shape of the face similar to the normal side by moving the angle of mouth tightly and adequately. Both esthetic and functional improvements can be achieved with the transfer of the temporalis tendon to the oral commissure.
